# Anorectal melanoma: systematic review of the current literature of an aggressive type of melanoma

**DOI:** 10.1097/CMR.0000000000001003

**Published:** 2024-10-04

**Authors:** Giovanni Paolino, Antonio Podo Brunetti, Carolina De Rosa, Carmen Cantisani, Franco Rongioletti, Andrea Carugno, Nicola Zerbinati, Mario Valenti, Domenico Mascagni, Giulio Tosti, Santo Raffaele Mercuri, Riccardo Pampena

**Affiliations:** a Unit of Dermatology, IRCCS Ospedale San Raffaele; b Università Vita-Salute, San Raffaele, Milan; c Dermatologic Unit, Department of Clinical Internal, Anesthesiological and Cardiovascular Sciences, La Sapienza University of Rome, Rome; d Department of Medicine and Surgery, University of Insubria; e Department of Medicine and Innovation Technology (DiMIT), University of Insubria, Varese; f Dermatology Unit, IRCCS Humanitas Research Hospital, Milan; g Department of Surgery, Sapienza University of Rome, Rome; h Dermato-Oncology Unit, IRCCS Istituto Europeo di Oncologia, Milan, Italy

**Keywords:** anal melanoma, anorectal, melanoma, rectal melanoma

## Abstract

Anorectal melanoma (ARM) is a rare malignancy often associated with a poor prognosis due to its late diagnosis and aggressive biological behavior. This review aims to comprehensively investigate ARM’s diagnosis, management, and treatment, emphasizing its clinical characteristics, laboratory findings, and implications for patient prognosis. A systematic literature search was conducted in PubMed, Embase, and Cochrane CENTRAL databases from inception to 1 July 2024. This review synthesizes existing literature to provide a comprehensive understanding of this rare primary malignancy. A total of 110 articles reporting on 166 patients were included. Gender data were available for 131 cases, comprising 67 females (51.1%) and 64 males (48.9%). The median age was 66 years. The overall median time to diagnosis was 4 months for anal melanoma, 3 months for rectal melanoma, and 4 months for anorectal junction melanoma. The clinical presentation was nodular in 98.2% of cases. Pre-diagnosis symptoms included bleeding in 84.9% of cases, mucous elimination (6%), pain (68.7%), tenesmus (16.9%), and changes in bowel movements (28.5%). Overall survival (OS) was reported in 82 cases, with a median OS of 11 months: 11 months for anal melanoma, 7 months for rectal melanoma, and 12 months for anorectal junction melanoma. ARM is a rare and aggressive melanoma subtype often diagnosed at an advanced stage, leading to a poor prognosis. A female predominance was observed, consistent with other mucosal melanomas. Anal melanoma exhibited better progression-free survival, and OS compared to rectal and anorectal junction melanoma.

## Introduction

Although melanoma usually manifests on the skin, in 0.8–3.7% of cases, it may also involve other anatomic locations such as mucous membranes, including the sinuses, nasal passages, oral cavity, vagina, anus, and rectum [1].

Anorectal melanoma (ARM) is a rare entity that corresponds to 1% of colorectal malignancies and less than 0.5% of anal canal malignancies [2], while ARM accounts for less than 1% of all melanoma cases and 16.5% of mucosal melanomas [3]. Due to its late diagnosis and biological behavior, ARM is often associated with a poor prognosis. Indeed, since tenesmus, pruritus, weight loss, lower gastrointestinal bleeding, and change in bowel habits are often associated with other common clinical manifestations of the anorectal area (such as hemorrhoids), the diagnosis is delayed. Besides, in 20–30% of cases, ARM is amelanotic, endoscopically resembling benign polypoid lesions [4], leading to a further misdiagnosis. Furthermore, the genetics of this malignancy are often different from the ones of cutaneous melanomas, with a rare v-raf murine sarcoma viral oncogene homolog B1 (BRAF) positivity and an increased presence of v-kit Hardy-Zuckerman 4 feline sarcoma viral oncogene homolog (c-KIT) and neuroblastoma rat sarcoma (RAS) viral oncogene homolog (NRAS) mutations.

Due to all these aspects, ARM’s staging, treatment, and management remain challenging. Therefore, a multidisciplinary approach is needed since the clinical manifestations of this neoplasm can be very different, and the related management can vary based on the experience of each specialist and Institute.

This review aims to comprehensively investigate and elucidate ARM’s diagnosis, management, and treatment, shedding light on its clinical characteristics, laboratory findings, and implications for patient prognosis. By systematically reviewing existing literature and consolidating relevant data, we seek to understand this rare primary malignancy comprehensively.

## Materials and methods

A systematic literature search was conducted from inception to 1 July 2024 in PubMed, Embase, and Cochrane CENTRAL databases. The following keywords were searched on PubMed: [‘anal melanoma’ (MeSH Terms)] OR [‘melanoma’ (All Fields) AND ‘anal’ (All Fields)] OR ‘anorectal’ (All Fields) OR ‘rectal’ (All fields). For Embase and Cochrane CENTRAL, the following terms were searched: (anorectal melanoma) and (anal melanoma) and (rectal melanoma). If needed, authors of the articles were also contacted, and reference sections were perused to identify all relevant reports and unpublished data. At first, we evaluated the entire sample and the related clinical-pathological characteristics. We analyzed the cases of ARM on the basis of the anatomical location, that is, whether anal, rectal, or anorectal junction, which correspond to the individual anatomical descriptions reported in the individual articles.

Only case reports, case series, clinical reports, and clinical trials in English have been included in the current analysis. For this review, we followed the Meta-analysis of Observational Studies in Epidemiology proposal and the Preferred Reporting Items for Systematic Reviews and meta-analysis guidelines, where feasible.

### Statistical analysis

Absolute and relative frequencies were calculated. Pearson *X*^2^ and Fisher exact tests were used for qualitative variables; quantitative variables were checked for normal distribution and compared via Kruskal–Wallis test.

Progression-free survival (PFS) was calculated from the diagnosis of melanoma to the date of the first metastatic event. In contrast, overall survival (OS) was calculated from the diagnosis of melanoma to the date of death or last follow-up. Kaplan–Meier survival plot was performed to estimate PFS and OS according to the specific anatomical site (anal, rectal, or anorectal junction). Patients who were lost to follow-up or who were alive at the time of the last follow-up were censored at the date of their last follow-up.

A multivariate backward stepwise Cox regression model was constructed, including gender, age, melanoma stage, and anatomic location. A *P* value <0.05 was considered statistically significant. The data were analyzed using the Statistical Package for the Social Sciences (SPSS) 29.0 (IBM Corp., Armonk, New York, USA).

## Results

### Study search and general demographic data

**Fig. 1:**
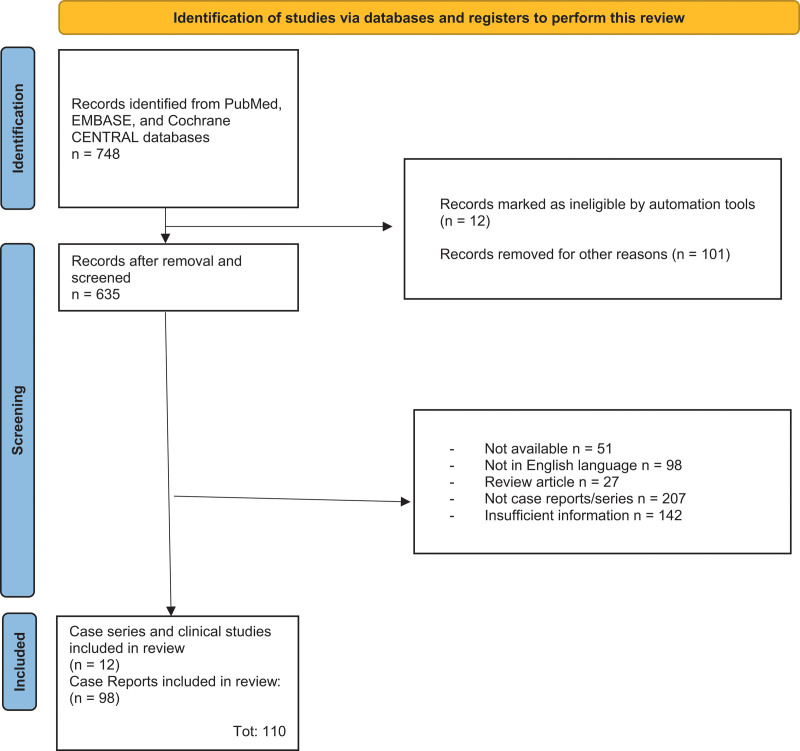
Flowchart of the search strategy.

The initial search of ARM retrieved a total of 748 studies. After removing duplicated studies and after removing records marked as ineligible by automation tools, 635 articles were screened according to the title/abstract, and subsequently 525 were excluded (Fig. 1). Specifically, 51 studies were not available, 98 studies were not in English language, 27 were review articles, 207 articles were not clinical cases (e.g. basic research, animal research), and in 142 articles there were insufficient information. Therefore, a final number of 110 [1,3,5–111] articles reporting 166 patients were included in this review, as summarized in Fig. 1 and Table 1, respectively. The included studies were published from 1988 to date, with a peak between 2004 and 2024 (85%; 94/110). Twelve (12/110; 11%) studies were case series, with 70 patients representing 42% of total patients and 17 patients reported in the more extensive study, while the remaining 98 studies were case reports (98/110; 89%). Regarding gender, 91 patients were female (54.8%), and 75 (45.2%) were male. The general median age was 66 years, and the range was between 20 and 88 years. The median time to reach diagnosis was 4 months, between 1 and 12 months. The clinical presentation of the lesions was nodular in 71% of cases (118/166). Regarding the anatomic localization of the primary melanoma, the rectum was involved in 54 cases (32.5%), anal in 27 cases (16.3%), and anorectal junction in 50 cases (30%); the anatomic site of the primary melanoma was missing for 35 cases (21.1%) (Table 1).

**Table 1:** Clinico-pathologic baselines

Variables	Anatomic location			Total	*P* value
	Anal	Rectum	Anorectal		
Age	66 (IQR 56–78)	67 (IQR 55–77.5)	65.5 (IQR 61.8–74)	66 (IQR 57–75.3)	0.79
Time to reach the diagnosis (months)	4 (IQR 3–5.3)	3 (IQR 2–6)	4 (IQR 2.8–7)	4 (IQR 3–6)	0.587
Gender					
Female	14	26	27	67	0.834
	51.9%	48.1%	54.0%	51.1%	
Male	13	28	23	64	
	48.1%	51.9%	46.0%	48.9%	
Total	27	54	50	131	
Other anorectal diseases					
No	24	41	43	108	0.335
	88.9%	77.4%	86.0%	83.1%	
Yes	3	12	7	22	
	11.1%	22.6%	14.0%	16.9%	
Total	27	53	50	130	
Clinical presentation					
Macular	0	1	1	2	0.727
	0.0%	2.1%	2.6%	1.8%	
Nodular	26	46	38	110	
	100.0%	97.9%	97.4%	98.2%	
Total	26	47	39	112	
Mucous elimination through anal canal					
No	24	51	48	123	0.45
	88.9%	94.4%	96.0%	93.9%	
Yes	3	3	2	8	
	11.1%	5.6%	4.0%	6.1%	
Total	27	54	50	131	
Blood elimination through anal canal					
No	3	8	8	19	0.799
	11.5%	14.8%	17.4%	15.1%	
Yes	23	46	38	107	
	88.5%	85.2%	82.6%	84.9%	
Total	26	54	46	126	
Pruritus					
No	26	53	49	128	0.858
	96.3%	98.1%	98.0%	97.7%	
Yes	1	1	1	3	
	3.7%	1.9%	2.0%	2.3%	
Total	27	54	50	131	
Pain					
No	20	37	33	90	0.766
	74.1%	68.5%	66.0%	68.7%	
Yes	7	17	17	41	
	25.9%	31.5%	34.0%	31.3%	
Total	27	54	50	131	
Tenesmus					
No	21	46	41	108	0.697
	77.8%	85.2%	83.7%	83.1%	
Yes	6	8	8	22	
	22.2%	14.8%	16.3%	16.9%	
Total	27	54	49	130	
Change in bowel movements					
No	20	38	35	93	0.931
	74.1%	71.7%	70.0%	71.5%	
Yes	7	15	15	37	
	25.9%	28.3%	30.0%	28.5%	
Total	27	53	50	130	
Inguinal masses					
No	26	50	43	119	0.878
	96.3%	94.3%	93.5%	94.4%	
Yes	1	3	3	7	
	3.7%	5.7%	6.5%	5.6%	
Total	27	53	46	126	
BRAF mutation					
No	3	9	6	18	0.622
	100.0%	90.0%	100.0%	94.7%	
Yes	0	1	0	1	
	0.0%	10.0%	0.0%	5.3%	
Total	3	10	6	19	
NRAS mutation					
No	2	2	1	5	0.301
	100.0%	100.0%	50.0%	83.3%	
Yes	0	0	1	1	
	0.0%	0.0%	50.0%	16.7%	
Total	2	2	2	6	
c-KIT mutation					
No	2	6	4	12	0.293
	100.0%	54.5%	40.0%	52.2%	
Yes	0	5	6	11	
	0.0%	45.5%	60.0%	47.8%	
Total	2	11	10	23	
Abdominoperineal resection^a^					
No	2	0	2	4	0.239
	10.0%	0.0%	5.1%	4.5%	
Yes	18	30	37	85	
	90.0%	100.0%	94.9%	95.5%	
Total	20	30	39	89	
Wide local excision^a^					
No	2	0	2	4	0.19
	25.0%	0.0%	18.2%	12.5%	
Yes	6	13	9	28	
	75.0%	100.0%	81.8%	87.5%	
Total	8	13	11	32	
Endoscopic mucosal resection^a^					
No	2	1	2	5	0.83
	28.6%	50.0%	40.0%	35.7%	
Yes	5	1	3	9	
	71.4%	50.0%	60.0%	64.3%	
Total	7	2	5	14	
Palliative local surgery^a^					
No	1	7	4	12	0.42
	100.0%	100.0%	80.0%	92.3%	
Yes	0	0	1	1	
	0.0%	0.0%	20.0%	7.7%	
Total	1	7	5	13	
Palliative colostomy for bowel obstruction^a^					
No	0	5	5	10	0.186
	0.0%	62.5%	71.4%	58.8%	
Yes	2	3	2	7	
	100.0%	37.5%	28.6%	41.2%	
Total	2	8	7	17	
Progression free survival					
	16 (IQR 3–51)	6 (IQR 3.8–14)	14 (IQR 6–26.5)	9 (IQR 4–22)	0.157
Progression free survival stat (0/1)					
No progression	4	6	5	15	0.908
	36.4%	35.3%	29.4%	33.3%	
Progression	7	11	12	30	
	63.6%	64.7%	70.6%	66.7%	
Total	11	17	17	45	
Overall survival	11 (IQR 3–30.8)	7 (IQR 5–13)	12 (IQR 7–23.5)	11 (IQR 5–21)	0.237
Overall survival stat (1/0)					
No death	8	14	19	41	0.885
	50.0%	46.7%	52.8%	50.0%	
Death	8	16	17	41	
	50.0%	53.3%	47.2%	50.0%	
Total	16	30	36	82	
Stage at time of diagnosis					
1	1	2	1	4	0.871
	6.3%	7.4%	3.7%	5.7%	
2	1	1	2	4	
	6.3%	3.7%	7.4%	5.7%	
3	5	9	13	27	
	31.3%	33.3%	48.1%	38.6%	
4	9	15	11	35	
	56.3%	55.6%	40.7%	50.0%	
Total	16	27	27	70	

BRAF, v-raf murine sarcoma viral oncogene homolog B1; c-KIT, v-kit Hardy-Zuckerman 4 feline sarcoma viral oncogene homolog; IQR, interquartile range; NRAS, neuroblastoma rat sarcoma (RAS) viral oncogene homolog.

a These data were evaluated based on subsets of patients. Evaluating the data according to the general population, surgery was the primary therapeutic approach in 105 (95%) patients, sometimes requiring multiple surgical approaches in the same patients, as reported in 20 cases (18%). Specifically, abdominoperineal resection was performed in 85 patients (76%), while a wide local excision was needed in 28 patients (25%). An endoscopic mucosal resection was performed only in nine cases (8%), while a palliative local surgery and a palliative colostomy for bowel obstruction were performed in one (0.9%) and seven (6%) cases, respectively.

Regarding systemic therapies, the treatments are multiple and different according to the year and period in which the study has been performed. Specifically, conventional chemotherapy was reported in 46 cases (temozololamide, dacarbazine, cis-platin), immunotherapy has been reported in 19 cases, interferon-alpha in 10 cases, and vemurafenib in 1 patient with a BRAF mutation. At the same time, radiotherapy has been reported in 23 cases. One patient was treated with an anti-vascular endothelial growth factor (VEGF) (apatinib). Knowles *et al*. reported four cases of ARM treated with tyrosine kinase inhibitor [112]. Multiple (sequential or combined) treatments have been performed in 12 cases.

### Melanoma clinic-pathologic baselines according to the anatomic area: anal, rectal, and anorectal junction

The gender was available in 131 cases, with 67 females (51.1%) and 64 (48.9%) males, although with some differences between anal melanoma, anorectal junction melanoma, and rectal melanoma, as reported in Table 2. The general median age of the cohort was 66 years. The overall median time to reach a diagnosis was 4 months for anal melanoma, 3 months for rectal melanoma, and 4 months for anorectal junction melanoma. The clinical presentation was nodular in 98.2% of cases. No patient had a previous history of cutaneous melanoma. The presence of other anorectal diseases was reported in 32 cases, with hemorrhoids as the most common associated anorectal disease (28/32; 87.5%). No patient reported a personal positive history of cutaneous melanoma, and no patient had an anamnesis positive for human papillomavirus or other anorectal infective diseases. Regarding the symptoms before the diagnosis of ARM, mucous elimination was reported in 6% of cases (8/131), while bleeding in 84.9% (107/126) of cases (Table 2). Other symptoms reported were pain (41/131; 68.7%), tenesmus (22/130; 16.9%), and changes in bowel movements (37/130; 28.5%) (Table 2).

**Table 2:** Clinico-pathologic features according to anatomic area if anal, rectum, or anorectal junction

Multivariate backward stepwise	PFS				OS			
	Hazard ratio	95% CI for hazard ratio		*P* value	Hazard ratio	95% CI for hazard ratio		*P* value
		Lower	Upper			Lower	Upper	
Age	1.041	0.994	1.091	0.089				
Stage								
4				ref.				ref.
1	0.082	0.007	0.938	0.044	0.347	0.044	2.716	0.313
2	0.392	0.070	2.205	0.288	0.000	0.000	.	0.981
3	0.230	0.060	0.889	0.033	0.195	0.073	0.516	<0.001
Anatomic location								
Anal				ref.				ref.
Rectum	7.758	1.431	42.049	0.018	3.855	1.138	13.068	0.030
Anal-rectum	0.842	0.224	3.170	0.800	1.583	0.500	5.014	0.435
Rectum				ref.				ref.
Anal	0.129	0.024	0.699	0.018	0.259	0.077	0.879	0.030
Anal-rectum	0.109	0.024	0.498	0.004	0.411	0.157	1.071	0.069
Dichotomic anal + anal-rectum ref.	8.701	2.025	37.388	0.004	2.828	1.150	6.959	0.024

CI, confidence interval; OS, Overall survival; PFS, progression-free survival.

At the time of diagnosis, the Prasad level was reported in 84 patients, with Prasad Level III as the most common (61/84; 72.6%). At the time of diagnosis, American Joint Committee on Cancer staging was reported in 70 cases, with stage IV as the most common (50%; 35/70). The other stages are reported in Table 2.

A BRAF mutation was reported in 5.3% of cases (1/19), N-RAS mutation in 16.7% (1/6), and c-KIT mutation in 47.8% of cases (11/23). Regarding the treatment, surgery was the primary therapeutic approach in 105 (95%) patients, sometimes requiring multiple surgical approaches in the same patients, as reported in 20 cases (18%). Specifically, abdominoperineal resection was performed in 85 patients (76%), while a wide local excision was needed in 28 patients (25%). An endoscopic mucosal resection was performed only in nine cases (8%), while a palliative local surgery and a palliative colostomy for bowel obstruction were performed in one (0.9%) and seven (6%) cases, respectively (for details, see Table 1 and Table 2).

### Survival analysis

PFS was reported in 45 cases, with a median PFS of 9 months, with 16 months for anal melanoma, 6 months for rectal melanoma, and 14 months for anorectal junction melanoma. OS was reported in 82 cases, with a median OS of 11 months, with 11 months for anal melanoma, 7 months for rectum melanoma, and 12 months for anorectal junction melanoma.

**Fig. 2:**
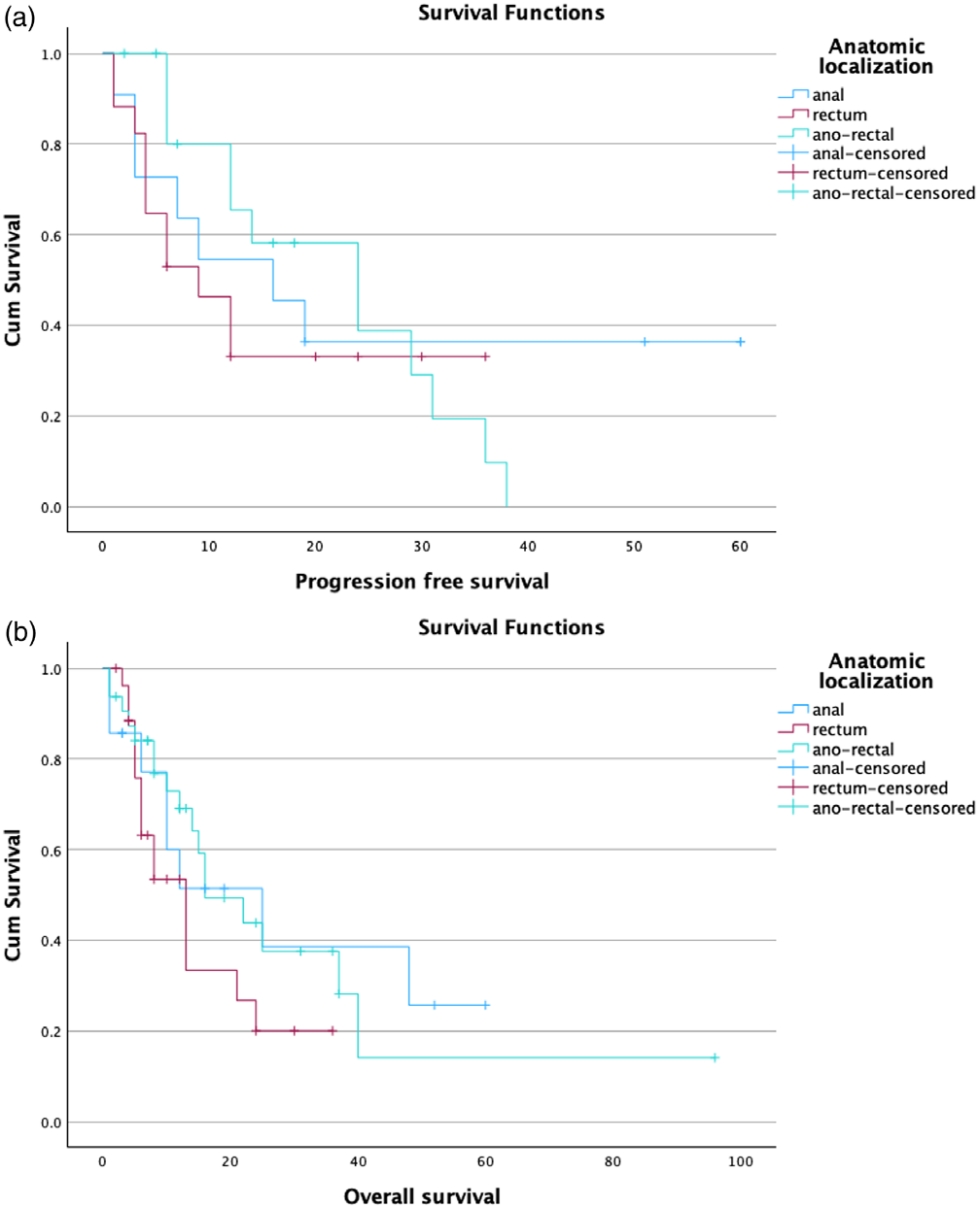
(a) Progression-free survival (PFS) according to anatomic areas. (b) Overall survival (OS) according to anatomic areas.

Kaplan–Meier curves showed no significant differences in anatomic locations concerning PFS and OS (Fig. 2). However, when including this variable in a Cox regression multivariate model together with age, gender, and melanoma stage, we observed a significantly higher risk of PFS for rectal melanoma as compared to both anal [hazard ratio: 7.8; 95% confidence interval (CI): 1.4–42.0; *P*: 0.018] and anorectal junction melanoma (hazard ratio: 9.2; 95% CI: 2.0–41.7; *P*: 0.004). Concerning OS, rectal melanoma showed a higher mortality rate than anal location melanoma (hazard ratio: 3.9; 95% CI: 1.1–13.1; *P*: 0.004). Finally, regarding the treatment, we did not find significance in terms of OS between patients who had undergone immunotherapy and patients who had not, with similar OS of 6 months and 8 months, respectively (*P* = 0.23).

## Discussion

Although fewer in number than in the skin, melanocytes can migrate to mucosal areas, where they play an antioxidant role [89]. Specifically, melanocytes can be found in the squamous zone of the anal canal and sometimes in the anal transitional zone [89]. However, these cells can undergo malignant transformation, leading to ARM. This malignant transformation may be induced by several factors, such as oxidative stress and immunosuppression, as well as ARM may originate from Schwannian neuroblastic cells of the autonomic intestinal innervation system or cells of the amine-precursor uptake and decarboxylation system of the gut [89,113]. ARM’s etiology remains to be discovered, with few investigations requiring further research.

Clinically, ARM usually presents as exophytic and polypoid lesions, often ulcerated and amelanotic, mimicking other anorectal tumors and diseases. Besides, symptoms induced by ARM are not specific, further delaying the diagnosis, with a high percentage of patients at the time of diagnosis already having a metastatic disease. The diagnosis is mainly histological and is usually characterized by epithelioid, spindle-cell, lymphoma-like, or pleomorphic malignant melanocytes [89]. Immunohistochemistry (S100+, Melan-A+, HMB-45+, and tyrosinase+) and somatic driver mutations in the c-KIT gene in about 75% of ARM may facilitate the diagnosis [114].

In our analysis, we found that the median age of the patients was 66 years, ranging between 20 and 88 years. Interestingly, we found a slight female prevalence, with 54.8% of cases. This higher incidence in women reflects the generally increased risk of developing mucosal melanoma in females than in male patients (2.8 vs. 1.5 per million), as reported by Mihajlovic *et al*. [115]. This reason may be associated with the higher presence of estrogen receptors in the mucosa of female patients than in males. However, other studies should further confirm this assertion [116,117].

The anal canal can be divided into three main zones: anus, rectum, and anorectal junction, and therefore, a primary ARM may arise in one of these three mucosal areas. Accordingly, we decided to investigate if ARM in one of these three anatomic areas may be associated with specific prognostic features. To the best of our knowledge, for the first time in literature, we found an increased PFS and OS statistically significant in anal ARM, compared to rectal ARM. This difference may be associated with the fact that anal melanoma can be diagnosed early compared to other anatomic areas, as well as this difference in terms of prognosis may be associated with the difference of the epithelium, with mucosal epithelium (present in the rectum and anorectal junction), following the biology of pure mucosal melanomas, with a worse prognosis. Indeed, we also found higher melanoma stages in the rectal and anorectal zone compared to the anal zone. Contrariwise, we have not found any significance regarding diagnostic timing to reach the diagnosis between these three anatomical areas.

PET is the most widely adopted modality for determination of local extension of the malignancy, visualizing perirectal lymph nodes and screening for distant metastasis to evaluate the patient status for therapeutic options [118,119]. The majority of ARMs at the time of diagnosis were classified as stage III or stage IV, with surgery and systemic therapies as the primary therapeutic options. In this regard, abdominoperineal resection was the primary surgical treatment, with local endoscopic surgery performed in minimal cases. Regarding systemic treatments, although to date there is no valid and official systemic treatment for ARM [118], it seems that combined therapies (e.g. ipilimumab/nivolumab or immunotherapy/radiotherapy or chemotherapy/radiotherapy) are the most reported treatments for this class of patients, as well as target therapy with tyrosine kinase inhibitors (such as imatinib or sorafenib) in patients with c-KIT mutation. Therefore, in ARM, combined treatments can improve the prognosis in this class of patients.

### Conclusion

ARM is a rare and aggressive type of melanoma, often associated with a worse prognosis since it is often diagnosed at an advanced stage with loco-regional or distant metastases. Usually, at the time of diagnosis, ARM presents as nodular or polypoid lesions, with c-KIT as the most common genetic mutation. As well as for the other forms of mucosal melanoma, we found a female prevalence. Anal melanoma shows a better prognosis in term of PFS and OS compared to rectal and anorectal junction melanoma. Surgery, with abdominoplasty resection, remains the first therapeutic option, while in advanced stages, systemic treatments are needed to increase PFS and OS. Unfortunately, immunotherapy itself does not appear to improve survival in ARM patients, while tyrosine kinase inhibitors can be effective in patients with c-KIT genetic mutation. Radiotherapy, traditional chemotherapy, and anti-VEGF treatments can also be taken into consideration since sometimes combined therapeutic options show an excellent therapeutic response.

## Acknowledgements

### Conflicts of interest

There are no conflicts of interest.
